# Unravelling inversions: Technological advances, challenges, and potential impact on crop breeding

**DOI:** 10.1111/pbi.14224

**Published:** 2023-11-14

**Authors:** Haifei Hu, Armin Scheben, Jian Wang, Fangping Li, Chengdao Li, David Edwards, Junliang Zhao

**Affiliations:** ^1^ Rice Research Institute, Guangdong Academy of Agricultural Sciences & Key Laboratory of Genetics and Breeding of High Quality Rice in Southern China (Co‐construction by Ministry and Province), Ministry of Agriculture and Rural Affairs & Guangdong Key Laboratory of New Technology in Rice Breeding & Guangdong Rice Engineering Laboratory Guangzhou China; ^2^ Simons Center for Quantitative Biology, Cold Spring Harbor Laboratory Cold Spring Harbor New York USA; ^3^ Guangdong Provincial Key Laboratory of Plant Molecular Breeding, State Key Laboratory for Conservation and Utilization of Subtropical Agro‐Bioresources South China Agricultural University Guangzhou China; ^4^ Western Crop Genetics Alliance, Centre for Crop & Food Innovation, Food Futures Institute, College of Science, Health, Engineering and Education Murdoch University Murdoch Western Australia Australia; ^5^ School of Biological Sciences University of Western Australia Perth Western Australia Australia; ^6^ Australia & Centre for Applied Bioinformatics University of Western Australia Perth Western Australia Australia

**Keywords:** inversion, pangenome, plant breeding, machine learning, genome editing

## Abstract

Inversions, a type of chromosomal structural variation, significantly influence plant adaptation and gene functions by impacting gene expression and recombination rates. However, compared with other structural variations, their roles in functional biology and crop improvement remain largely unexplored. In this review, we highlight technological and methodological advancements that have allowed a comprehensive understanding of inversion variants through the pangenome framework and machine learning algorithms. Genome editing is an efficient method for inducing or reversing inversion mutations in plants, providing an effective mechanism to modify local recombination rates. Given the potential of inversions in crop breeding, we anticipate increasing attention on inversions from the scientific community in future research and breeding applications.

## The importance and progress of studies on inversions

Inversions are DNA regions that have changed in orientation and represent an important category of genomic structural variations (SVs). While inversions represent a relatively small percentage of SVs across different organisms ranging from 0.5% to 7% (Chaisson *et al*., 2019; Zhang *et al*., 2023; Zhao *et al*., 2016), they can span over 100 Mb and collectively account for up to 10% of the genome (Walkowiak *et al*., 2020), having significant functional and evolutionary impacts on species. The classification of inversions remains an open question, with many analyses defining classes based on arbitrary absolute lengths, making comparisons between species of varying genome sizes difficult. A potentially more comparable and functional classification of inversions into three groups can be conducted based on their lengths relative to the chromosome size: small (>50 bp and <0.0025%), intermediate (≥0.0025% and <1.25%) and large inversions (≥1.25%). These guideline percentages are evaluated based on commonly used inversion length thresholds (Bansal *et al*., 2007; Harringmeyer and Hoekstra, 2022; Zhang *et al*., 2023; Zhou *et al*., 2023), with the hope that further studies will enable a more rigorous functional classification of inversions. Inversions with different lengths tend to have different evolutionary and functional impacts on organisms. Particularly, intermediate or large inversions can suppress local recombination, facilitating the selection of adaptive traits, promoting reproductive isolation, and leading to speciation (Hoffmann and Rieseberg, 2008; Huang and Rieseberg, 2020b).

Inversions were first identified in *Drosophila* species through the comparison of genetic linkage maps (Sturtevant, 1921) and further studied in the human genome using the G‐banded karyotype technique (Yunis and Prakash, 1982). Subsequent studies have shown inversions to be widespread across plants (Jin *et al*., 2023; Li *et al*., 2022; Wang *et al*., 2023), human (Chaisson *et al*., 2019; Ebert *et al*., 2021) and animals (da Silva *et al*., 2019; Li *et al*., 2021). Inversions are prevalent in plants, representing a significant yet often enigmatic source of genome evolution. For example, Chen *et al*. (2013) estimated that about 15.7–32.1 inversions occur approximately once every million years based on 214 inversions between AA and BB *Oryza* species. Recently, Zhou *et al*. (2023) estimated an inversion rate of 67.4 inversions per million years using 1769 inversions, which is two to four times higher than previous estimations. The observed differences may arise from the differential capacity to identify inversions of varying sizes between the two studies, likely attributable to the accessibility and quality of the genomes used.

Inversions play a pivotal role in driving genome evolution and are integral to the evolutionary processes of numerous species (Faria *et al*., 2019b; Todesco *et al*., 2020; Wellenreuther and Bernatchez, 2018). Inversions can exert direct functional effects through their breakpoints and indirectly facilitate positive selection by capturing beneficial allele combinations and preventing recombination from breaking them up (Connallon and Olito, 2022). For instance, suppressed recombination among genes within an inversion can lead to largely independent genome evolution between derived and ancestral arrangements, providing opportunities for divergence and speciation (Faria *et al*., 2019b). This genomic isolation, facilitated by inversions, can lead to the formation of novel genotypes and phenotypes, driving the genetic diversity we observe today.

Besides their evolutionary significance, studies of inversions also shed light on crop breeding applications. Chromosomal inversions can modify the recombination frequency of genes during meiosis, altering trait inheritance (Stevison *et al*., 2011). They may induce complications during fertilization and embryonic development, resulting in decreased fertility or offspring with genetic abnormalities. Therefore, discerning the frequency and distribution of inversions in populations is important for breeding programs aimed at enhancing or fixing the inheritance of traits (Jay *et al*., 2018). Inversions can also serve as genetic markers to monitor the inheritance of specific traits or to identify related individuals within breeding programs (Jonah *et al*., 2011). A comprehensive map of inversion polymorphisms within germplasm will provide breeders with a valuable reference, enabling them to use knowledge of this structural variation in the breeding process. Moreover, inversions can affect the gene expression associated with important agronomic and adaptive traits, by reorganizing large regulatory domains (Naseeb *et al*., 2016), modifying genetic or epigenetic environments near their breakpoints (Wesley and Eanes, 1994), and preserving linkage with regulatory elements within or near the inverted region due to suppressed recombination in heterozygotes (Lavington and Kern, 2017).

Before the advent of high‐throughput sequencing technologies, inversion studies were confined to methods, such as cytogenetics or PCR‐based approaches. However, these methods are time‐consuming, costly, and suffer from limited resolution, hindering the detection of many inversions in complex genomic regions and on a population scale. Consequently, the study of inversions was restricted to a select number of well‐characterized model organisms. In the past decade, facilitated by advancements in genomic technologies, such as high‐throughput next‐generation sequencing (NGS) and third‐generation sequencing, studies of inversions have been increasingly investigated across multiple species (Stein *et al*., 2018; Yuan *et al*., 2021). The advent of next‐generation DNA sequencing technologies has greatly transformed the field of genomics, enabling the detection and characterization of inversions in diverse organisms, including non‐model species (Niedzicka *et al*., 2016). Moreover, NGS has paved the way for large‐scale population studies of inversions, enabling the discovery of polymorphic inversions and their association with phenotypic traits. With the help of long‐read DNA sequencing, the study of inversions was further accelerated with increasingly high‐quality whole‐genome data and high‐quality genome assemblies becoming available. Recently, the development of artificial intelligence, such as machine learning, which can identify complex patterns and relationships in data, can significantly enhance the accuracy of inversion detection and trait association (Wu *et al*., 2020; Zhou *et al*., 2023). This progress has fostered a more profound understanding of the evolutionary and functional significance of inversions across different organisms.

## Inversion detection methods

Cytological methods, DNA marker‐based methods, and sequence‐based methods are three approaches to inversion detection, each with its own distinct advantages and disadvantages (Table 1).

**Table 1:** Advantages and shortcomings of methods to detect inversions

Inversion detection methods	Specific methods	Pros	Cons	Detectable inversion
Cytological methods	FISH‐based chromosome painting	High sensitivity and specificity, allow for the visualization of chromosomal structures	Cannot capture population‐wide inversions since FISH only works for specific known sequences and may miss small inversions due to its limited resolution	Intermediate‐to‐large inversions
	Optical mapping	Effective in identifying large inversions and provides long‐range information about the genome	Does not provide actual sequence information, is expensive and requires specialized equipment and software for data analysis	Intermediate‐to‐large inversions
	G‐banding cytogenetics	Simple, cost‐effective and allows visual observation of chromosomal inversions	Cannot capture population‐wide inversions, is time‐consuming, effect relies on sample preparation, and is unable to detect small inversions	Intermediate‐to‐large inversions
DNA marker‐based methods	Genetic linkage maps, linkage disequilibrium/local population structure, outlier signals detection	Provides a broad overview of inversion distribution and their relative positions, information about recombination hotspots and suppressed areas related to inversions using short‐read sequencing data	Time‐consuming and require a large population for genetic linkage map construction and only can detect inversions that affect recombination. Can not pinpoint the precise inversion breakpoints	Intermediate‐to‐large inversions
Sequence‐based methods	Short read sequencing	Detects inversions at a relatively high resolution from a large sample size with effective cost	Has a high rate of false positives and negatives on inversion detection due to short read length and is difficult to detect inversions in complex regions	Small‐to‐intermediate inversions
	Long read sequencing	Long‐read sequencing can span the entire length of an inversion and provide a detailed view of inversion breakpoints	The cost of long‐read sequencing is relatively high for the generation of population‐scale sequencing data	Small‐to‐large inversions
	Hi‐C sequencing	Can provide high‐resolution maps of chromosomal interactions and 3D information to help inversion identification	Detecting inversions solely based on Hi‐C data can be ambiguous as changes in chromatin conformation or nuclear organization can also lead to contact frequency change	Small‐to‐large inversions

### Cytological methods

Early studies of inversions relied on cytogenetic analyses of chromosomes, where inversions could be inferred from variations in chromosome banding patterns observed in karyotypes or studies of chromosome pairing during meiosis (Anderson *et al*., 2010; Rodriguez *et al*., 2000). Fluorescent *in situ* hybridization (FISH) based chromosome painting is one of the most widely used cytogenetic methods for identifying chromosome structural variations and has been applied for inversion discovery in several plant species (Braz *et al*., 2020; de Oliveira Bustamante *et al*., 2021; Szinay *et al*., 2012). Optical mapping, which involves *in vitro* restriction enzyme digestion of DNA sequences together with the labeling, and imaging of extended DNA molecules, represents another highly efficient method for detecting structural variation including inversions (Levy‐Sakin and Ebenstein, 2013). This approach can visually represent inversions through a conspicuous reversal of expected sequence positions, making a significant step forward from traditional cytogenomic techniques, such as G‐banding cytogenetics and Fibre FISH. Despite its efficacy, optical mapping can be prone to false negatives in inversion detection, requiring supplementary technologies to enhance precision and accuracy.

### DNA marker‐based methods

Advances in DNA molecular genetic marker technology, including the construction and comparison of genetic linkage maps and population structure analysis, enabled the detection of chromosomal inversions within and across species (Doganlar *et al*., 2002). For example, when the genetic linkage maps of pepper and tomato were compared, 19 inversions and 6 chromosome translocations were observed between these two genomes, along with several potential single‐gene transpositions (Wu *et al*., 2009). Furthermore, examining linkage disequilibrium (LD) or local population structure across the genome with tools, such as the R package lostruct (Li and Ralph, 2019), and identifying outlier signals can identify potential inversions, leveraging the impact of inversions on population structure. Using this approach, inversions in sunflowers were detected *de novo*, and their frequencies in natural populations were determined (Faria *et al*., 2019a; Huang *et al*., 2020a). Drawbacks of such marker‐based methods include that their resolution and detection accuracy are limited by marker density and population genetic factors such as recombination rate.

### Sequence‐based methods

Traditional cytogenetics and population DNA marker‐based methods are labor‐intensive and lack resolution, which can limit their effectiveness. The development of more efficient tools for detecting and characterizing inversions is essential to improve our understanding of inversion polymorphisms in populations. NGS technologies provide an efficient tool for inversion identification. Several strategies have been used for detecting chromosomal inversions based on short‐read sequencing, including:

- Paired‐end mapping: This technique maps NGS reads to a reference genome assembly and identifies inversions by finding read pairs that map in an orientation inconsistent with the expected orientation based on the fragment size (Medvedev *et al*., 2009). Using this strategy, we developed an efficient pipeline (PSVCP) for capturing and identifying inversions within a pangenome (Wang *et al*., 2023). Notably, inversions longer than 300 bp (longer than the longest short reads) become harder to detect via short‐read sequencing.
- Split‐read mapping: This approach identifies reads spanning an inversion breakpoint and aligns the two segments of the read to different regions of the reference (Rausch *et al*., 2012). Similarly, the ability to detect larger inversions using short‐read sequencing is limited as they are too long covered by short reads.
- Assembly‐based methods: These methods involve the *de novo* assembly of short reads into scaffolds or high‐quality chromosome scale assemblies that can help identify the presence of inversions through comparison of these assemblies with a reference genome. Inversions are identified when the orientation of the DNA sequence contradicts the expected orientation based on the reference genome (Goel *et al*., 2019; Zapata *et al*., 2016). However, it is easy to misassemble genetic regions with unnormal GC content or abundant repeat regions using short reads, bringing difficulties in accurately identifying inversions based on the comparison between genome assemblies.

Long‐read DNA sequencing including PacBio HiFi sequencing, Oxford Nanopore duplex sequencing and ultra‐long sequencing enables the generation of longer sequencing reads (10 kb–2 Mb) to address the limitations of short reads for detecting inversions. Identifying inversions becomes considerably straightforward with the use of long reads that can span the repetitive and complex genomic regions. High‐quality genome assembly or even Telomere‐to‐Telomere (T2T) genome assembly produced using long reads can serve as a gold‐standard reference to create an inversion index database (Zhou *et al*., 2023) or a graph‐based genomic framework (Zhou *et al*., 2022), allowing efficient mapping and genotyping by short‐read sequences.

Other sequencing technologies, such as chromosome‐conformation capture (Hi‐C) sequencing, can also be used to predict three‐dimensional (3D) genome structure and identify chromosomal inversions, offering the potential to define chromosome breakpoints down to base pair resolution (Spielmann *et al*., 2018). However, the 3D structure of the genome can vary between different cell types, and therefore the results of Hi‐C experiments may not be representative of all cell types in an organism. Therefore, the integration of Hi‐C with other techniques, such as long‐read sequencing or optical mapping, can enhance the accuracy and resolution of inversion detection.

## Current status of inversion studies in plants

Recently, several inversion studies have been conducted on crops and fruit plants, including rice (Qin *et al*., 2021; Stein *et al*., 2018; Wang *et al*., 2018, 2023; Zhou *et al*., 2023), soybean (Liu *et al*., 2020), barley (Jayakodi *et al*., 2020; Zhang *et al*., 2023), wheat (Walkowiak *et al*., 2020), brassica (Boideau *et al*., 2022; Cai *et al*., 2021), cotton (Jin *et al*., 2023; Ma *et al*., 2021), tomato (Wang *et al*., 2020) and cucumber (Li *et al*., 2022) (Table 2). These studies highlighted that inversions have a significant impact on environmental adaptation and domestication in plants, by modifying recombination rate, gene expression and linkage disequilibrium. For example, Wang *et al*. (2020) identified 28 inversions ranging from 483 bp to 13.9 Mb between two cultivated tomato genomes and found that more than half of the genes located within inversions were functionally associated with biotic and abiotic resistance. Further genotyping of these 28 inversions across 597 diverse tomato accessions, including wild, landrace, modern, and other cultivars, revealed lower allele frequencies of 17 inversions in cultivated tomatoes compared to wild tomatoes, suggesting a shift during domestication. Moreover, another study in barley demonstrated that domesticated barley accessions from northern European origins contain a 10 Mb inversion on chromosome 2H (Jayakodi *et al*., 2020). These accessions carry the *HvCEN* haplotype III, which is associated with later flowering time and adaptation to northern European climates. Besides, our recent work identified another 9 Mb inversion on chromosome 2H that is present specifically in the Australian barley population, harbouring genes functioning in salt, drought and temperature stress responses (Hu *et al*., 2023).

**Table 2:** Summary of current plant and crop inversion studies

Species	Genome number and population size	Inversion number and size	Detection method	Whether using a pangenome framework	Key findings	References
Rice	73 genomes and 3 K‐RGP data set	1769 (0.3–15 Mb)	Genome comparison and genotyping by NGS and machine learning	Yes	Inversions influence gene expression, recombination rate, and linkage disequilibrium	Zhou *et al*. (2023)
Rice	3 K‐RGP	152 ± 62 per genome (127.1 ± 19.4 kb)	Read mapping and detection of inversion breakpoints using NGS	Yes	A large percentage of inversions (37.9%) occur differentially between the *indica* and *japonica* subpopulations	Wang *et al*. (2018)
Rice	13 genomes	9 paracentric inversions (60–300 kb)	Genome comparison	No	Paracentric inversions may contribute to the rapid diversification of AA genome species	Stein *et al*. (2018)
Rice	12 genomes and 413 lines from the RPD panel	3326 (100 bp–129 kb)	Genome comparison and genotyping by NGS	Yes	Chromosome 6 harbours the greatest number of inversions, and genes within these inversions are predominantly associated with rice blast disease resistance	Wang *et al*. (2023)
Rice	33 genomes	954 (100 bp–5 Mb)	Genome comparison	Yes	Most *O. sativa* accessions and *O. glaberrima* exhibit the same state for a Chromosome 6 inversion identified between the *indica* and *japonica* subpopulations	Qin *et al*. (2021)
Maize	26 genomes	35 (>1 Mb)	Genome comparison	Yes	Inversions occur in regions comprising 49.8% fewer genic base pairs compared to the overall genomic background	Hufford *et al*. (2021)
Wheat	16 genomes	139 (11–153 Mb)	Genome comparison and Hi‐C analysis	No	Several pericentric inversions result in shifts of the position of the centromere on chromosomes 4B and 5B	Walkowiak *et al*. (2020)
Soybean	26 genomes	3120 (100 kb–>1 Mb)	Genome comparison	Yes	Inversions emerged during domestication and showed differences between wild and cultivated soybean varieties	Liu *et al*. (2020)
*Brassica rapa*	16 genomes	736–2479 (5.93–14.47 Mb)	Genome comparison	Yes	A ~ 1.3 Mb inversion specifically occurred in the Chiifu and CCB Chinese cabbage genomes	Cai *et al*. (2021)
*Brassica napus*	9 genomes	131–239 (1–19.46 Mb)	Genome comparison and optical maps or oligo‐FISH analysis	No	Inversions occur frequently between *B. napus* species and may contain key agronomic genes such as disease resistance genes	Boideau *et al*. (2022)
Barley	20 genomes and 300 diverse barley lines	42 (4–141 Mb)	Genome comparison and Hi‐C analysis	Yes	Large inversions can suppress recombination and may associate with local environment adaptation during barley expansion of growing geographical range	Jayakodi *et al*. (2020)
Cultivated barley	7 genomes	222 (500 bp–17.1 Mb)	Genome comparison and Hi‐C analysis	No	A 9 Mb inversion showing regionally specific occurrence in Australia may affect genes closest to the inversion breakpoint that functions in salt, drought and temperature stress response	Hu *et al*. (2023)
Wild barley	3 genomes	225 (620 bp–37.12 Mb)	Genome comparison and Hi‐C analysis	No	Large chromosome inversions may result in a heterogeneous pattern of genomic differentiation	Zhang *et al*. (2023)
Cotton	4 genomes	243–9515 (140 bp–1.77 Mb)	Genome comparison	No	Changes in gene function may occur when inversions intersect with the exons of genes	Ma *et al*. (2021)
Cotton	11 genomes	2236 (2 kb–32 Mb)	Genome comparison	Yes	Inversion can lead to a strong suppression of recombination rates	Jin *et al*. (2023)
Cucumber	11 genomes	9–42 (<478 Kb)	Genome comparison and Hi‐C analysis	Yes	An inversion map offers a roadmap for the selection of parental lines when establishing segregating populations between wild and cultivated cucumbers	Li *et al*. (2022)
Tomato	2 genomes and 597 tomato accessions	28 (483 bp–13.9 Mb)	Genome comparison and Hi‐C analysis	No	A large percentage of genes harboured by inversions were functionally associated with disease resistance and response to abiotic stress	Wang *et al*. (2020)
Sunflower	1506 wild sunflowers from 3 species	9 (3.5–100.8 Mb)	Genome comparison, genetic maps alignment and Hi‐C analysis	No	Haploblocks linked with inversions can suppress recombination and maintain adaptive allelic combinations	Todesco *et al*. (2020)
Sunflower	1445 individuals from 3 sunflower species	9 (3.5–100.8 Mb)	Genome comparison, genetic maps alignment and Hi‐C analysis	No	The load of mutations in sunflower inversions is inversely related to the heterozygosity of the inversions	Huang *et al*. (2022)
Sunflower	120 accessions	9 (11–57 Mb)	Local population structure analysis and outlier discovery method	No	An adaptive divergence of a dune sunflower ecotype is facilitated by the presence of several chromosomal inversions	Huang *et al*. (2020a)

The rapid advancement of DNA sequencing technologies, coupled with declining sequencing costs, has enabled the construction of genome assemblies for a wide range of species. However, a reference genome built from a single individual falls short of representing the genetic diversity within a species, due to inherent genetic variations between individuals (Bayer *et al*., 2020; Danilevicz *et al*., 2020; Golicz *et al*., 2020) and pangenomes have been constructed for several crop species (Bayer *et al*., 2022b; Golicz *et al*., 2016; Rijzaani *et al*., 2022; Yu *et al*., 2019; Zhao *et al*., 2020). Therefore, a pangenome supplemented with a consistent chromosome coordinate framework is required to capture the complete genetic variations of a plant species from a collection of genetic sequences of diverse cultivars and their crop wild relatives. This strategy has been implemented in several research studies. For example, Zhou *et al*. (2023) captured the genetic diversity of Asian rice by investigating 73 rice genomes, which included two wild rice accessions. They constructed an inversion index within a pangenome framework, containing 1769 non‐redundant inversions. Employing this pangenome‐wide coordinate system, they successfully characterized the distribution of inversions and identified hotspots across both wild and cultivated Asian rice varieties, uncovering 885 *O. rufipogon* and 96 *O. punctata* specific clustered inversions. Similarly, another study employing 33 rice genomes used a SV‐based pangenome framework approach, revealing that most *O. sativa* accessions and *O. glaberrima* share the same state for a chromosome 6 inversion that was detected between the *Indica* and *Japonica* subpopulations (Qin *et al*., 2021).

Recent advances in the field of pangenomics have led to the development of graph‐based pangenomes (Edwards and Batley, 2022). In the graph‐based pangenome framework, genetic variants are represented as nodes and edges, thereby maintaining sequence continuity and accurately representing structural variations between individuals (Eizenga *et al*., 2020). While many early plant pangenomes focused on variation in gene content (Montenegro *et al*., 2017), more recent pangenomes (Bayer *et al*., 2022a) have been graph‐based due to the increasing availability of high‐quality genome assemblies (Zanini *et al*., 2021). Bioinformatics tools for constructing SV graphs, such as Minigraph‐Catus (Hickey *et al*., 2023) and PGGB (Garrison *et al*., 2023), facilitated the presentation of haplotypes of the inversion allele among 15 individuals at the ch2L:17 144 069 position in the *D. melanogaster* pangenome. Similarly, through the construction of a pangenome‐wide inversion graph, Li *et al*. (2022) provided a strategy for selecting parental lines during the establishment of segregating populations between wild and cultivated cucumbers.

Although long‐read sequencing can now generate telomere‐to‐telomere assemblies (Rautiainen *et al*., 2023) that render detection of most inversions trivial, the lower cost of short‐read sequencing will continue to make it an attractive approach for genotyping inversions at population scale. However, detecting inversions using short‐read sequencing data are challenging, as the state of inversion breakpoints covered by sequencing reads can vary, and inversions may even be surrounded by duplications or other types of structural variations within the inversion regions. These challenges can be further compounded in polyploid or heterozygous plants, where different haplotypes or subgenomes can carry different inversion alleles.

To address the limitations of short reads, machine learning, due to its proficiency in feature mining, has been incorporated into inversion identification algorithms to enhance the accuracy and sensitivity of detection. Machine learning has been applied to support gene annotation (Upadhyaya *et al*., 2022), assess genome structural variation (Bayer *et al*., 2021) and link genomic variation with crop traits (Danilevicz *et al*., 2021; Gill *et al*., 2022). DeepVariant is a powerful machine learning tool using a deep neural network (DNN) to detect and genotype small genetic variants, such as SNPs and INDEls (Poplin *et al*., 2018), but showing a limited ability to detect inversions. Machine learning applications specifically for inversion studies include an inversion discovery tool InvBFM, which is developed to extract and mine inversion features from high‐throughput short‐read sequencing data using a support vector machine (SVM) classifier (Wu *et al*., 2020). The authors demonstrated that their inversion classifier surpassed previous read alignment‐based inversion calling methods, such as Delly (Rausch *et al*., 2012) and Lumpy (Layer *et al*., 2014). Similarly, Zhou *et al*. (2023) implemented a machine‐learning workflow to genotype inversions across high‐throughput sequencing data from the 3K‐Rice Genome Project, providing insights into species formation from the perspective of inversion events throughout evolutionary history. One limitation of these methods is that there is limited empirical training data, because inversions are relatively rare and plant genome sequencing efforts remain limited in scale. A solution may thus be to train inversion detection methods using simulated SVs (Dierckxsens *et al*., 2021). In the future, to optimally leverage these machine learning methods for inversion genotyping with short reads or long reads in plants, we believe thousands of haplotype‐resolved assemblies generated by long‐read sequencing will be a powerful asset for computing genotype ‘truth’ sets, generating aligned training data and tuning models.

## Advances in genomics and genome editing pave the way for inversion‐based plant breeding

**Figure 1:**
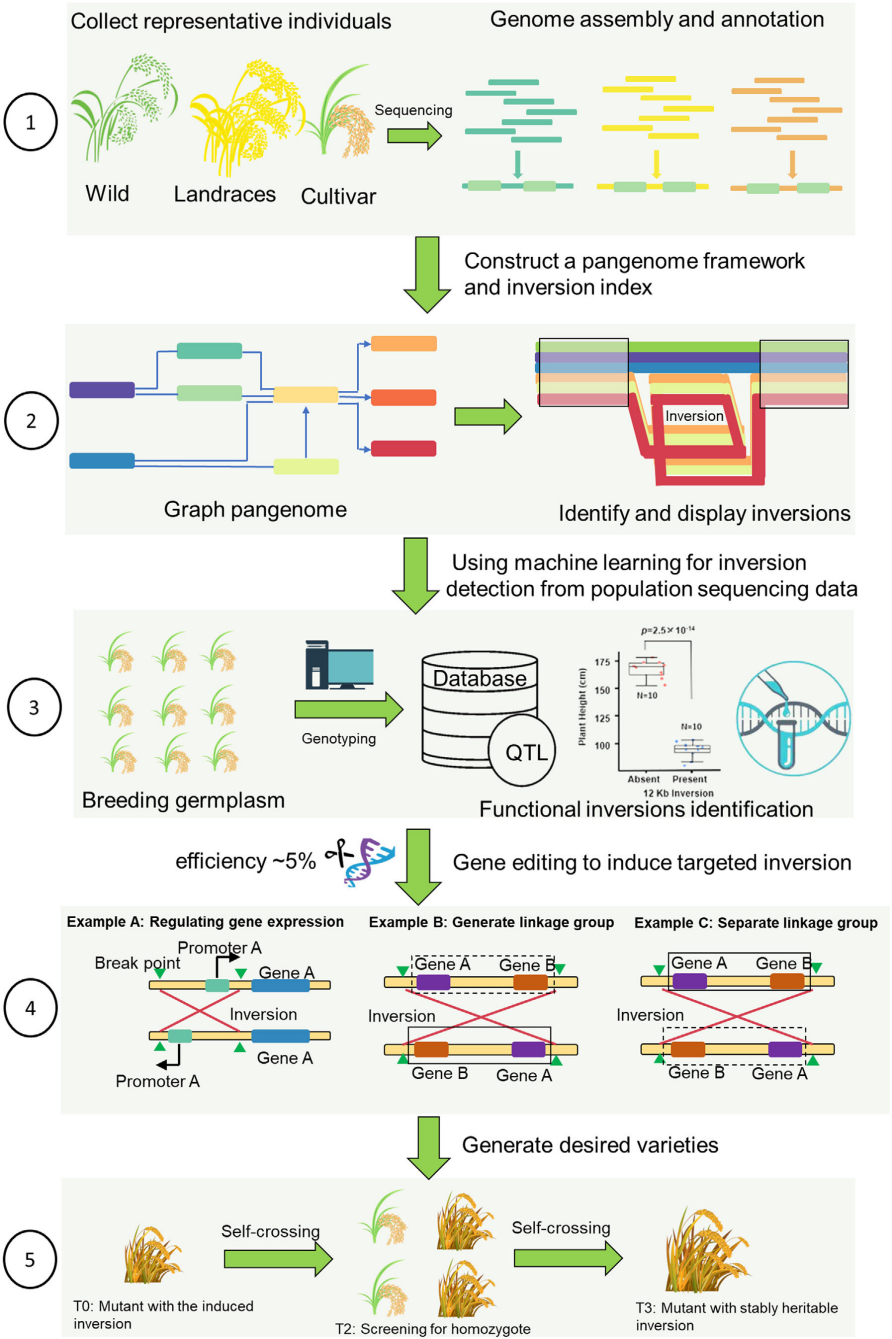
Key stages in the proposed breeding workflow integrating new technologies include a pangenome framework, machine learning and gene editing for crop improvement and designing new varieties with desired traits. Step 1: Collect multiple individuals of the plant species of interest from a broad range of geographic and ecological conditions to capture as much genetic diversity as possible; obtain genome sequences of these selected samples and assemble them into high‐quality reference genomes and perform gene annotation to characterize gene structure and identify non‐coding regions. Step 2: Construct a graph‐based pangenome and identify the structural variations including inversions in a pangenome index; apply machine learning algorithms to detect the inversions from a large scale of breeding germplasm data. Step 3: To identify the functional inversions, search the overlap inverted regions with QTL mapping with agronomic traits, perform the association analysis between inversion haplotype and phenotype and experimentally validated the candidate inversions. Step 4: Three examples showing how to use the CRISPR/Cas genome editing to induce the heritable and functional inversions for crop improvement (a successfully induced rate of inversion: ~5%). The induced inversion can modify the orientation of the promoter and will turn off the transcription of Gene A (Example A). Given that the recombination between reshuffled sections of the genome is hindered in heterozygotes, we can create new linkage groups by inducing an inversion. When two unconnected genes reside at various places on the chromosome arm, they are likely to be split up during the formation of crossover in meiosis. However, if an inversion that includes both genes is induced, they can be linked to ensure they are inherited as a pair (Example B). Conversely, previously established inversions can be reversed and thereby break up linkage groups caused by such inversions (Example C). Step 5: Generation of a stable mutant crop line from T0 to T2 involves initial genetic modification of T0 plants, followed by self‐fertilization to produce T1 and then T2 generations. If a T1 plant is homozygous for the mutation, all its T2 offspring will be homozygous, indicating stable inheritance of the mutation.

Increasing evidence supports the proposition that large inversions can serve as valuable genetic resources for crop breeding and viable target sites for CRISPR‐Cas editing (Ronspies *et al*., 2021; Scheben *et al*., 2017; Tay Fernandez *et al*., 2022). This is due to the role inversions play in suppressing the recombination by inhibiting the crossing over of chromosome segments, as well as their potential to affect the expression of genes associated with biotic and abiotic resistance. Thus, we propose an inversion‐based plant breeding pipeline that integrates a pangenome framework and machine learning approaches with genome editing techniques to induce or modify heritable inversions (Figure 1). The pipeline begins with generating, assembling, and annotating long‐read genome sequences from diverse individuals of a plant species into high‐quality reference genomes. Using a graph‐based pangenome framework, non‐redundant inversions and their patterns across genomes specific to lineages or ecotypes are detected using a pangenome index. Subsequently, it is possible to genotype the targeted inversions at the population level using high‐throughput sequencing data along with machine learning algorithms and perform inversion haplotype and phenotype association analysis to evaluate their effect on phenotypic variation. Inversions that disrupt gene structure or regulatory elements, or that overlap with Quantitative Trait Loci (QTL) associated with agronomic traits, are further classified as candidate functional inversions that may have a significant impact on crop breeding.

To engineer desirable inversions, two double strand breaks (DSBs) are induced on the same chromosome using genome editing (Lee *et al*., 2011). Repair of the DSBs then usually occurs via classical non‐homologous end joining (NHEJ) (Schmidt *et al*., 2019), which can produce random chromosomal rearrangements such as inversions. This method induces inversions in the same way as they can spontaneously occur in the wild. However, achieving precise inversions can be challenging because inversions occur at low rates and are often associated with deletions at the inversion junctions (Schmidt *et al*., 2019; Schwartz *et al*., 2020). These unwanted deletions co‐occurring with inversions can disrupt genes or regulatory elements. Progress has been made in overcoming these technical hurdles and generating seamless inversions at higher rates. Unsurprisingly, a rate limiting factor for rearrangements like inversions is the rate of induced DSBs (Ronspies *et al*., 2022). Thus, maximizing nuclease cutting efficiency is critical for achieving high inversion rates. By tuning the Cas9‐driving promoter and the nuclease cutting efficiency in the target region, inversions without indels can be efficiently induced in wild‐type plants at rates of 0.5% (Schmidt *et al*., 2019) and in some cases even up to 10% (Schmidt *et al*., 2019). Although these low to moderate efficiencies can still be a hurdle for small‐scale research studies, they are sufficient for applications in plant breeding.

Genome editing studies have demonstrated induced inversions in the model plants Arabidopsis (Ronspies *et al*., 2022; Schmidt *et al*., 2019; Zhang *et al*., 2017), rice (Lu *et al*., 2021) and maize (Schwartz *et al*., 2020). Zhang *et al*. (2017) successfully generated inversion mutations in sequences containing exon regions of a pair of homologous flowering locus genes with opposite functions, Arabidopsis FLOWERING TIME (*AtFT*) and TERMINAL FLOWER 1 (*AtTFL1*), altering its flowering time. In maize, genome editing was used to re‐invert a 75.5 Mb pericentric genomic region in an elite inbred line (Schwartz *et al*., 2020), breaking up the linkage of genes previously locked in the inverted region and allowing recombination with other inbred lines. The functional consequences of these inversions on recombination rates have also been studied, showing that crossing over can be induced in a recombination cold spot by reverting a natural 1.1 Mb inversion in Arabidopsis (Schmidt *et al*., 2020). Conversely, an induced 17 Mb inversion in Arabidopsis suppressed crossing over in the region (Ronspies *et al*., 2022). These studies inducing large inversions up to 75 Mb also suggest that the physical size of induced inversions is not a limiting factor. Finally, in a different application of inversion engineering, Lu *et al*. (2021) induced a 911 kb inversion in rice to increase expression of the target gene *PPO1* over ten‐fold by swapping its promoter with the promoter of the downstream gene *CP12*.

## Concluding remarks

Inversions can have a major impact on gene expression, recombination, and linkage disequilibrium, and thus significantly influence environmental adaptation and domestication in plant species. Recent technological advances and methodological developments have enabled the comprehensive characterization of genetic diversity and the elucidation of inversion variants through pangenome indices. Furthermore, machine learning algorithms have proven effective in mining inversion features, thereby improving the accuracy of inversion detection at the population level using inexpensive short‐read sequencing. Additionally, the application of genome editing techniques represents an efficient approach for inducing heritable inversions. However, despite these technological strides, studies on inversions continue to face numerous challenges. Techniques used to identify and genotype inversions have not kept up with methods focusing on other types of structural variants, such as deletions and duplications. This disparity arises in part because inversions are copy‐neutral. Unlike copy‐number variants, they do not result in changes to the depth of coverage of sequence reads, thereby increasing the complexity of inversion detection. The graph‐based pangenome framework, although promising, is still in the early stages of development, and robust tools for the population‐level analysis and visualization of graph‐based pangenomes remain limited. Furthermore, plants containing complex repeat regions can confuse aligners and impede the construction of a graph‐based pangenome, particularly for polyploid crops such as wheat and oat with large genome sizes. In addition, a significant challenge of employing machine learning algorithms to detect inversions is the lack of feature training data, given that inversions are relatively rare compared to other types of structural variations, and more benchmarked and validated inversions within a species are needed to improve the accurate mining and detection of inversion features. Moreover, while genome editing techniques hold promise, their efficiency in targeting inversions remains low, and modified Cas9 proteins are required to efficiently induce targeted inversions for functional studies and crop improvement.

The study of inversions and their potential functions in plants is important but remains largely unexplored. The ongoing advances in technologies across disciplines, such as pangenomics, machine learning, and genome editing, are paving the way toward a deeper understanding of the biological and functional roles of inversions. The importance of inversions deserves more attention in the future, given that this significant genetic variant holds an underestimated potential for breeding better crops.

## Conflict of interest

The authors have not declared a conflict of interest.

## Author contributions

All authors co‐wrote the manuscript.
